# Standardized *Artemisia annua* Exhibits Dual Antileishmanial Activity and Immunomodulatory Potential In Vitro

**DOI:** 10.3390/vetsci12100950

**Published:** 2025-10-01

**Authors:** Estefania Morua, Laura Cuyas, Carlos J. Bethencourt-Estrella, Atteneri López-Arencibia, Maria Garrido Martínez, Ana Sañudo Otero, Jacob Lorenzo-Morales, José E. Piñero, Anabel Yetano Cunchillos, Raquel Virto Resano, Luis Matías-Hernández

**Affiliations:** 1 Biotech Tricopharming Research, 08018 Barcelona, Spain; em@tricopharming.com (E.M.);; 2 Institute of Tropical Diseases and Public Health of the Canary Islands, University of La Laguna, 38200 San Cristóbal de la Laguna, Spain; 3 Department of Obstetrics and Gynecology, Pediatrics, Preventive Medicine and Public Health, Toxicology, Legal and Forensic Medicine, and Parasitology, University of La Laguna, 38200 San Cristóbal de la Laguna, Spain; 4 Biomedical Research Networking Center on Infectious Diseases (CIBERINFEC), Instituto de Salud Carlos III, 28220 Madrid, Spain; 5 National Center for Food Technology and Safety (CNTA), 31570 San Adrian, Spain

**Keywords:** *Artemisia annua extract*, artemisinin, canine leishmaniasis, cytokine modulation, immunomodulation, *Leishmania infantum*

## Abstract

Leishmaniasis is a parasitic disease caused by *Leishmania* spp., transmitted by sandflies, and endemic in 98 countries. *Leishmania infantum*, the main agent of visceral leishmaniasis in Europe, commonly infects both humans and animals, with dogs as the principal domestic reservoir. Clinical manifestations in dogs depend on the host immune response. A robust Th1 response facilitates macrophage activation and parasite control, while persistently elevated TNF-α and IL-6 can lead to chronic inflammation and tissue damage. Current treatments reduce parasite load but rarely achieve complete cure and are often associated with relapses and resistance. *Artemisia annua*, source of artemisinin, could be a promising alternative to canine leishmaniasis. Despite its potential, no published studies have investigated its effect specifically against *Leishmania infantum* as well as its possible dual action: antiparasitic and immunomodulation. We conducted in vitro evaluations of a standardized *Artemisia annua* extract. Leishmanicidal activity was assessed against both promastigote and amastigote stages, and cytokine modulation was evaluated in RAW 264.7 macrophages. The extract showed strong leishmanicidal activity without cytotoxicity and significantly reduced TNF-α and IL-6 levels under inflammatory conditions, and in both cases, efficiency was correlated with artemisinin content. These results support *Artemisia annua* as a promising safer therapeutic adjuvant candidate for canine leishmaniasis, targeting both the parasite and the host inflammatory response.

## Simple Summary

Leishmaniasis, a parasitic disease transmitted by sandflies, affects both humans and animals, with dogs serving as the primary domestic reservoir of *Leishmania infantum*. Current treatments are often limited by relapses, adverse effects and the emergence of resistance. This study aimed to evaluate the in vitro antileishmanial and immunomodulatory effects of *Artemisia annua* extracts against *Leishmania infantum*. Extracts of *Artemisia annua* were tested on parasites and immune cells to assess antiparasitic efficacy, modulation of inflammatory responses, and cytotoxicity. *Artemisia annua* extracts exhibited strong activity against the parasite and effectively reduced harmful inflammatory responses in host immune cells, without inducing toxicity. Moreover, these dual therapeutic effects showed a clear dose-dependent relationship with artemisinin concentration within the extracts. As artemisinin increases, so does the magnitude of both therapeutic effects. These findings support artemisinin-rich *Artemisia annua* as a promising plant-based complementary strategy for veterinary use against *Leishmania infantum* infection, with preliminary indications of a favorable safety profile in vitro and potential translational value in leishmaniasis control.

## 1. Introduction

Canine Leishmaniasis (CanL) is a vector-borne disease caused mainly by the kinetoplastid protozoa *Leishmania infantum*, endemic in over 90 countries with an estimated 150 million dogs infected globally, including 2.5 million in Europe [1,2,3]. Climate change and global mobility are increasing its incidence by facilitating sandfly spread into temperate regions and facilitating the introduction of infected dogs beyond endemic areas [4,5,6].

Transmission occurs mainly via sandfly bites, though non-vector routes such as sexual, vertical, and blood transfusions also exist. Infected dogs exhibit a wide range of symptoms, often driven by immune dysregulation [4,7]. Cats can also harbor and transmit the parasite, usually showing milder symptoms unless immunosuppressed [8].

Control relies on prevention (insecticidal collars, vector avoidance, vaccination) [9]. Insecticide collars, with their insect-repellent properties, reduce the number of sandfly bites and consequently lower the percentage of dogs becoming infected. Vaccination, on the other hand, acts more effectively once the protozoan is present by stimulating the immune system, thereby reducing the severity of clinical symptoms. Current vaccines elicit protective Th1 immune responses, reducing incidence by 68–80%, though some animals remain susceptible and antibodies induction due to vaccination can complicate serological diagnosis due to false positives [10].

Treatment with drugs such as meglumine antimoniate, miltefosine, and allopurinol rarely achieves complete cure; and relapses within 5–12 months are common [11]. Chronic disease management often involves long-term use, which can trigger adverse effects (e.g., nephrotoxicity, inflammation, urolithiasis) and drug resistance, especially to allopurinol [12,13,14]. These challenges emphasize the urgent need for novel immunotherapeutics without adverse effects, and more effective alternatives, including natural product-based strategies.

*Artemisia annua* (also known as qinghao in Chinese), is a medicinal plant from the *Asteraceae* family with over 2000 years of therapeutic use in Traditional Chinese Medicine. Its most notable breakthrough lies in the discovery of its antimalarial compound, artemisinin [15]. Artemisinin contains a natural endoperoxide bridge that, upon activation, generates cytotoxic reactive oxygen species (ROS) [16,17]. Given *Leishmania’s* limited ROS defenses, the parasite is especially vulnerable to oxidative damage [18].

Artemisinin has shown promising efficacy against diverse *Leishmania* species in vitro, ex vivo, and in vivo such as *Leishmania donovani*, *Leishmania major*, *Leishmania amazonensis*, *Leishmania braziliensis*, *Leishmania mexicana, Leishmania tarentolae*, *Leishmania infantum* and *Leishmania tropica* [19,20,21,22,23]. Flavonoids, camphor, β-caryophyllene, and β-caryophyllene oxide may synergize with artemisinin, contributing to increased leishmanicidal activity [24,25,26,27]. These findings suggest that the complex phytochemical profile of *Artemisia annua* may act through complementary mechanisms reinforcing the promise of whole-plant formulations against *Leishmania* species. Despite the importance of *Leishmania infantum* in dogs, its susceptibility to *Artemisia annua* has not yet been tested, representing a gap in current research.

T-cell mediated immunity plays a central role in determining the clinical outcome of *Leishmania infantum* infection. In companion animals, disease progression depends on the balance between Th1 and Th2 pathways: Th1-mediated response is associated with protection and control, and the Th2-mediated response is linked to disease progression [28,29]. Th1 responses (IFN-γ, IL-2, TNF-α) activate macrophages and nitric oxide (NO) production, leading to parasite clearance [9,28,30].

Recent advances in immunopharmacology highlight the potential of plant-derived compounds to modulate immunity. *Artemisia annua* and its principal active compound, artemisinin, show ability to modulate both innate and adaptive immunity by enhancing macrophage function, reducing oxidative stress, and regulating T-cell responses [26,31,32,33,34,35]. In leishmaniasis models, artemisinin restores macrophage NO production and boosts IFN-γ and IL-2, reinforcing Th1 activity [36,37]. Some studies also report stronger IFN-γ responses suggesting that the immune system is leaning more towards a Th1-type response, even though some Th2 activity is still present [21,38].

Studies using whole-plant extracts of *Artemisia annua* have yielded similar or even superior results compared to artemisinin alone. Leaf extracts increased IFN-γ while reducing IL-4 and IL-10 in mice infected with *Leishmania donovani*, suggesting a synergistic action of the plant’s bioactive compounds that enhances artemisinin’s immunomodulatory effects [26].

While Th1 cytokine stimulation is essential for parasite clearance, controlling pro-inflammatory mediators is equally important to prevent immune-mediated damage. IL-6 is associated with increased disease severity in chronic leishmaniasis, as it suppresses IFN-γ production and enhances B-cell activation, contributing to hypergammaglobulinemia and systemic damage. Similarly, TNF-α, although essential for macrophage activation, may lead to tissue injury and immune exhaustion when overexpressed [39,40]. Thus, an effective response requires not only Th1 stimulation but also balanced cytokine regulation to avoid chronic inflammation. Dysregulated pro-inflammatory activity may compromise Th1 efficacy, fostering infection persistence.

Given *Leishmania infantum*’s relevance in dogs and its accelerated spread through climate change and migration, *Artemisia annua* emerges as a promising natural product-based alternative. This study aims to evaluate its antileishmanial activity against *Leishmania infantum* and explore mechanisms by which it may beneficially modulate host immunity.

## 2. Materials and Methods

### 2.1. Plant Material

Dried leaf powder of *Artemisia annua* was used for all experimental procedures. The plant material was obtained from *Artemisia annua* cultivated at our experimental site in Tenerife, Canary Islands (Spain), under open-field conditions and without synthetic pesticides or chemical fertilizers. Fresh leaves were harvested at diverse development stages, air-dried at 40 °C for 24 h, ground using a mechanical mill and sieved to obtain a homogenized fine powder suitable for experimental use.

### 2.2. Quantification of Artemisinin Content

The artemisinin content in the powdered *Artemisia annua* leaf material was quantified using ultra-high-performance liquid chromatography coupled with tandem mass spectrometry (UHPLC-MS/MS). A total of three distinct extracts were evaluated in the leishmanicidal assays, with artemisinin concentrations (mean ± SD from three independent analyses) of 1.21 ± 0.07%, 0.75 ± 0.02%, and 0.13 ± 0.01%, respectively. For the immunomodulatory experiments, at least two separate extracts were tested, containing 0.60 ± 0.02% and 0.89 ± 0.02% artemisinin.

The quantification method was adapted from the protocol originally developed for HPLC analysis [41], and optimized for UHPLC-MS/MS, employing a triple quadrupole mass spectrometry detector.

Chromatographic separation was performed on an Acquity UPLC^®^ HSS T3 column (100 × 2.1 mm, 1.8 µm particle size), complemented by a matching guard column. Sample extraction was carried out by suspending 10 mg of dried, ground, and sieved *Artemisia annua* leaf powder in 1 mL of acetone:water (75:25, *v*/*v*). The mixture was vortexed for 1 min, sonicated for 5 min, and centrifuged at 4000 rpm for 5 min at 20 °C. The extraction procedure was repeated once, and the supernatants were pooled prior to analysis.

All solvents and analytical reagents were of HPLC grade. Acetone (Sigma-Aldrich, St. Louis, MO, USA, Ref. 34850-M; EMD Millipore, ≥99.8%) and Milli-Q^®^ ultrapure water were used for extraction. Quantification was performed using certified HPLC-grade artemisinin standards (Sigma-Aldrich, St. Louis, MO, USA, Ref. 69532).

### 2.3. In Vitro Activity Assays Against the Promastigote Stage of Leishmania infantum

The activity of the compounds was tested in vitro against the promastigote stage of *Leishmania infantum* using a colorimetric assay based on Alamar Blue^®^ reagent (Invitrogen, Life Technologies, Madrid, Spain). Three different *Artemisia annua* extracts containing 1.21%, 0.75%, and 0.13% artemisinin, respectively, were evaluated. The extracts were first dissolved in dimethyl sulfoxide (DMSO) at 20 mg/mL, then serially diluted in RPMI 1640 medium (Gibco, Life Technologies, Madrid, Spain) using agitation and ultrasound to ensure complete homogenization, making sure that no more than 2% DMSO was added per well to avoid toxicity. Prior to testing, all solutions were filtered through 0.22 μm pore membranes to ensure sterility. Serial dilutions of the compounds were prepared in 100 μL of RPMI 1640 medium without phenol red and supplemented with 10% heat-inactivated fetal bovine serum in 96-well plates. The assay began with a starting concentration of 1 mg/mL per well, followed by ten serial dilutions down to 1.95 μg/mL.

Parasites in logarithmic growth phase were counted, diluted to 10^5^ cells/well, and added to each well. Subsequently, 10% Alamar Blue^®^ reagent was added, and the plates were incubated at 26 °C. After 72 h, parasite viability was assessed using an EnSpire^®^ Multimode Plate Reader (Perkin Elmer, Madrid, Spain), with absorbance measured at 570 nm and 630 nm as reference. Two independent experiments were conducted for the 0.75% and 1.21% artemisinin extracts, while only one experiment was performed for the 0.13% extract.

### 2.4. Activity Assays Against Intracellular Leishmania infantum Amastigotes

To assess the antileishmanial activity of the compounds against the intracellular amastigote stage, 50 µL of J774A.1 (ATCC TIB-67) macrophages were seeded into 96-well culture plates at a density of 2.5 × 10^5^ cells/mL (https://www.atcc.org/search#q=TIB-67&sort=relevancy&numberOfResults=24, accessed on 1 July 2025). J774A.1 (ATCC TIB-67) macrophages were cultured in RPMI, supplemented with 10% FBS at 37 °C and 5 % CO_2_ atmosphere. Cells were subsequently infected with 50 µL of *Leishmania infantum* metacyclic promastigotes (~6-day-old culture) at a parasite-to-host cell ratio of 10:1. The infected cultures were incubated overnight at 37 °C in an atmosphere containing 5% CO_2_ to promote parasite internalization.

After incubation, non-internalized promastigotes were removed by thorough washing with RPMI 1640 medium. The infected macrophages were then treated with either fresh medium (untreated control) or serial dilutions of the test compounds, following the same protocol used for the promastigote assays. Cultures were incubated for 24 h under standard conditions (37 °C, 5% CO_2_).

To assess parasite viability, infected macrophages were lysed using a modified protocol based on Jain et al. (2012) [42]. Briefly, plates were first washed with serum-free RPMI 1640, and the medium was carefully removed. Then, 30 µL of RPMI 1640 containing 0.05% SDS was added to each well, followed by gentle shaking for 30 s to lyse the host cells. Subsequently, 170 µL of RPMI 1640 supplemented with 10% fetal bovine serum were added to support parasite recovery. To detect viable parasites, 10 µL of AlamarBlue^®^ reagent were added to each well. The plates were then incubated at 26 °C to facilitate the differentiation of surviving amastigotes into promastigotes. After 72 h, parasite viability was determined using an EnSpire^®^ Multimode Plate Reader (Perkin Elmer, Madrid, Spain), measuring fluorescence at an excitation wavelength of 570 nm and emission at 585 nm. All data are representative of three biologically independent experiments.

### 2.5. Determination of IC_50_

The IC_50_ values were calculated using the Quest Graph™ IC_50_ Calculator (AAT Bioquest, Inc., Pleasanton, CA, USA; https://www.aatbio.com/tools/ic50-calculator, accessed on 31 July 2025), which employs a four-parameter logistic (4PL) regression model. Calculations were performed using raw activity data obtained from the previously described promastigote and amastigote assays to evaluate the concentration of each compound required to reduce parasite viability.

### 2.6. Citotoxicity Assay in Macrophages

A cytotoxicity study was performed using the murine macrophage cell line J774A.1 to evaluate the safety profile of the tested products. Macrophages were cultured in RPMI 1640 medium supplemented with 10% fetal bovine serum (FBS) and maintained at 37 °C in a 5% CO_2_ atmosphere. Cells were seeded in 96-well plates at a concentration of 2 × 10^5^ cells/mL (50 µL/well) and allowed to adhere for 2–4 h. The assay was initiated by exposing the macrophages to each extract starting at 400 µg/mL, followed by serial dilutions. Test solutions were prepared in deep-well plates and added to the adherent cells along with 10% AlamarBlue^®^ reagent. After 24 h of incubation under standard culture conditions, fluorescence was measured at 544 nm excitation and 590 nm emission using an EnSpire^®^ Multimode Plate Reader (PerkinElmer, Madrid, Spain). The fluorescence intensity was directly proportional to the metabolic activity of the viable macrophages. Data shown are based on three independent measurements.

### 2.7. Cell Culture, Maintenance and Cytotoxicity Assessment by MTT Assay

RAW 264.7 (ATCC TIB-71) cell line was kindly provided by Soria Natural (https://www.atcc.org/search#q=TIB-71&sort=relevancy&numberOfResults=24, accessed on 1 July 2025). RAW 264.7 cells were cultured in DMEM (Ref. 10569, Gibco, Thermo Fisher Scientific, Waltham, MA, USA) supplemented with 10% heat-inactivated fetal-bovine serum (Ref. 10500064 Gibco, Thermo Fisher Scientific, Waltham, MA, USA) without antibiotics. Cells were passaged three times per week using a scrapping method.

Cell viability assays were performed to determine the optimal concentration of *Artemisia annua* extracts for immune response studies. Cell viability was evaluated using the MTT assay (Sigma Aldrich, St. Louis, MO, USA, M2128), based on the reduction in the yellow, water-soluble tetrazolium salt MTT (3-(4,5-dimethylthiazol-2-yl)-2,5-diphenyltetrazolium bromide) to an insoluble, blue formazan product by the mitochondrial enzyme succinate dehydrogenase. The level of enzymatic activity correlates directly with mitochondrial function and, consequently, with cell viability.

Two types of aqueous extracts were prepared using 1 g of dried leaf material from *Artemisia annua*. For the acetone-based extract, the plant material was subjected to two successive extractions with 100 mL of acetone. After solvent evaporation under reduced pressure and subsequent drying, a concentrated extract was obtained, which was then re-dissolved in water at a fixed solid-to-liquid ratio (250 mg leaves/250 mL). Similarly, for the ethanol-based extract, the same procedure was followed, using absolute ethanol instead of acetone. The material was extracted twice with 100 mL of ethanol, and the solvent was evaporated and dried to yield a concentrated extract, which was also re-dissolved in water using the same solid-to-liquid ratio. The ethanol-based extract contained 0.6% artemisinin, while the acetone-based extract contained 0.9%.

RAW 264.7 macrophages were seeded into 96-well plates and incubated for 24 h to allow cell adherence. Subsequently, the test extract was added, and cells were incubated for an additional 24 h. After treatment, MTT solution was added to each well, followed by a 4 h incubation at 37 °C. The resulting formazan crystals were dissolved using DMSO, and absorbance was measured at 570 nm using a microplate reader.

### 2.8. Evaluation of the Extract on the Immune Response

IL-6 and TNF-α immune response in vitro assays were performed in collaboration with the National Center for Food Technology and Safety (CNTA), Spain, using the RAW264.7 murine macrophage cell line, a well-established model for studying immune activation and inflammation. The method involves inducing a cellular inflammatory response in RAW264.7 macrophages using lipopolysaccharide (LPS, Sigma Aldrich, St. Louis, MO, USA, L8274) from *Escherichia coli* O127:B8.

Cytokines released into the culture medium are analyzed using the ELISA technique (Enzyme-Linked ImmunoSorbent Assay). Hydrocortisone at 100 μM (Sigma Aldrich, St. Louis, MO, USA, Ref. H0888), known for its anti-inflammatory properties, was used as a positive control and added simultaneously with the test samples. Each sample was analyzed in triplicate, and at least three independent experiments were performed to ensure reproducibility. The experimental workflow was as follows: RAW 264.7 macrophage cell suspension was seeded into 96-well plates and incubated for 24 h. The test extract was then added, and cells were incubated for an additional 3 h. Afterward, LPS was added to stimulate the immune response, and cells were incubated 6 h further. Following incubation, the supernatants were collected after centrifugation. Cytokine quantification was performed using the ELISA immunoassay technique (Invitrogen, Carlsbad, CA, USA, Ref. 88-7064, 88-7324).

### 2.9. Statistical Analysis

Data are presented as mean ± standard deviation (SD). Statistical significance was determined using Student’s *t*-test, with results considered significant at *p* < 0.05 compared to the LPS control. All analyses were performed using Microsoft Excel (Microsoft Office, version 2019).

## 3. Results

### 3.1. In Vitro Activity of Artemisia annua Extracts Against Leishmania infantum Promastigotes

Promastigotes of *Leishmania infantum* are the flagellated, extracellular form of the parasite, enabling transmission by the sandfly vector and initiating infection in the mammalian host. The effect of *Artemisia annua* extracts on *Leishmania infantum* promastigotes was studied by evaluating parasite viability after 72 h of incubation. Three *Artemisia annua* different extracts containing varying concentrations of artemisinin (0.13%, 0.75%, and 1.21%) were tested. The assay was performed using serial dilutions, starting at 1 mg/mL and decreasing to 1.95 μg/mL.

**Figure 1:**
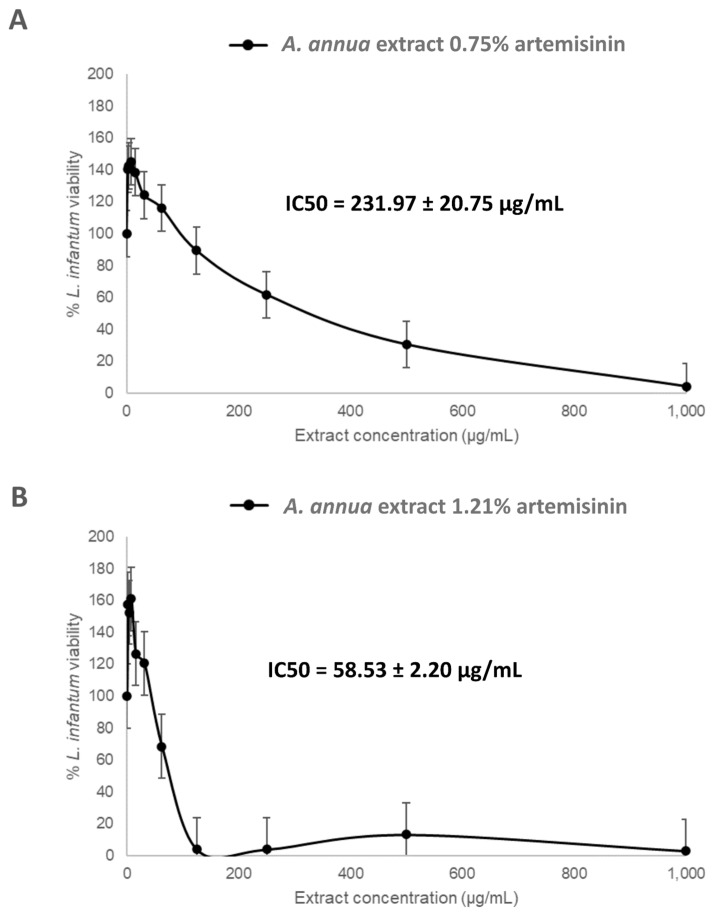
Anti-promastigote activity of *Artemisia annua* leaves. Log-phase *Leishmania infantum* promastigotes were incubated for 72 h at 27 °C with two *Artemisia annua* extracts containing 0.75% (**A**), and 1.21% (**B**) artemisinin, respectively. Promastigote viability was assessed by a colorimetric assay using Alamar Blue^®^, following exposure to serial dilutions of each extract (1 mg/mL to 1.95 μg/mL). IC_50_ values were determined for each extract. Each point represents the mean ± SD of two independent experiments.

Parasite viability was measured through a fluorescence-based assay and expressed as a percentage relative to untreated controls. As shown in Figure 1 and Supplementary Figure S1A the results revealed a clear artemisinin-dose-dependent inhibition of parasite viability across the three extracts. The extract with the lowest artemisinin content (0.13%) showed minimal inhibitory activity, with a shallow curve and an IC_50_ value of 782.25 µg/mL, indicating poor potency even at the highest concentrations tested (Supplementary Figure S1A). In contrast, the extract containing 0.75% artemisinin exhibited moderate antiparasitic activity, with a defined sigmoidal curve and an IC_50_ of 231.97 ± 20.75 µg/mL (Figure 1A). Notably, the extract with the highest artemisinin concentration (1.21%) displayed a strong inhibition profile, reaching near-complete parasite clearance at concentrations below 200 µg/mL. This extract achieved a lower IC_50_ of 58.53 ± 2.20 µg/mL (Figure 1B). Overall, these data indicate a direct correlation between artemisinin content and antileishmanial efficacy, with the 1.21% extract emerging as a promising candidate for further testing due to its superior activity profile.

### 3.2. Activity of Artemisia annua Extracts Against Leishmania infantum Amastigotes

The most effective extract against *Leishmania infantum* promastigotes, the *Artemisia annua* extract containing 1.21% artemisinin, was further evaluated against the intracellular amastigote stage, which represents the clinically relevant, replicative form of the parasite residing within host macrophages. To recover amastigotes, a controlled lysis of infected J774A.1 murine macrophages was performed, followed by incubation under previously described conditions to allow their transformation back into promastigotes for viability assessment.

Parasite viability was determined as previously described using the AlamarBlue^®^ assay. Infected cultures were treated with six serial dilutions starting at 250 and decreasing to 15.625 µg/mL. The assay was performed in three independent biological experiments, and both the percentage of inhibition and the IC_50_ value were calculated. The resulting IC_50_ was 69.32 ± 12.79 µg/mL, confirming the extract’s inhibitory activity against the intracellular stage.

### 3.3. Cytotoxicity of Artemisia annua Extracts in Murine Macrophages

**Figure 2:**
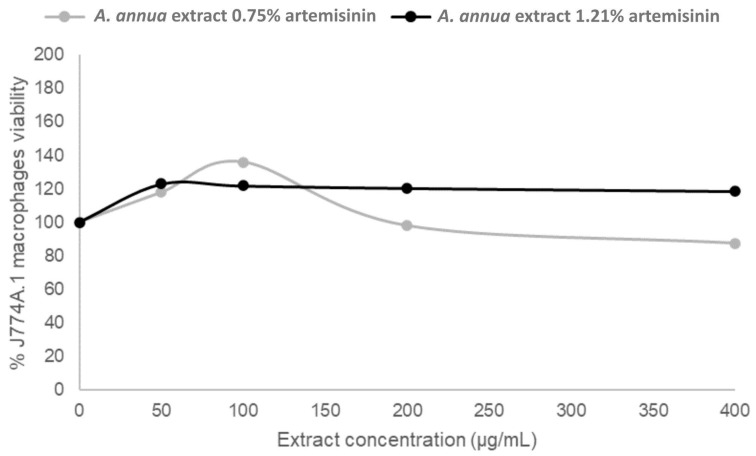
Cytotoxicity of *Artemisia annua* extract in uninfected J774A.1 macrophages. Cytotoxicity assay, with two *Artemisia annua* extracts containing 0.75% and 1.21% artemisinin, on uninfected J774A.1 macrophages treated with increasing concentrations of the same extract (50 to 400 µg/mL) for 24 h. Cell viability was measured using AlamarBlue^®^ and remained above 90% at all tested concentrations.

Cytotoxicity assays were performed on uninfected J774A.1 murine macrophages to evaluate the safety profile of the *Artemisia annua* extracts. Each extract, containing 0.13% (Supplementary Figure S1B), 0.75%, or 1.21% artemisinin (Figure 2), was tested starting at an initial concentration of 400 µg/mL, followed by three serial dilutions (200, 100, 50, and 0 µg/mL as control) to assess potential dose-dependent effects on cell viability. After 24 h of incubation, cell viability was measured using a fluorescence-based assay (Figure 2, Supplementary Figure S1B).

Across all concentrations tested, including the highest dose of 400 µg/mL, none of the extracts exhibited cytotoxic effects, with macrophage viability consistently remaining above 90%. These results indicate that the *Artemisia annua* extracts are non-toxic to host cells and demonstrate good selectivity toward the parasite, particularly the highly active 1.21% artemisinin preparation.

### 3.4. Evaluation of the Immunomodulatory Effect of Artemisia annua Extracts: Impact on IL-6 Production

Artemisinin and its derivatives have been shown to modulate key signaling pathways involved in immune cell activation, including the MAPK, NF-κB, and CDK cascades [43,44]. To further investigate these immunomodulatory effects, we conducted a series of in vitro assays using *Artemisia annua* extracts with two distinct artemisinin concentrations (0.6% and 0.9%), aiming to evaluate their impact on the production of pro-inflammatory cytokines, specifically IL-6 and TNF-α. These cytokines are frequently elevated in chronic inflammatory conditions and play critical roles in host defense, particularly in the clearance of intracellular pathogens.

Interleukin-6 (IL-6) is one of the most prominent pro-inflammatory cytokines, involved in both protective immunity and pathological inflammation. It is a multifunctional mediator that plays a pivotal role in the acute-phase response and in the differentiation and activation of immune cells, particularly T lymphocytes and B cells. Elevated IL-6 levels are indicative of systemic inflammation and are commonly observed in intracellular parasitic infections titus [45,46,47].

**Figure 3:**
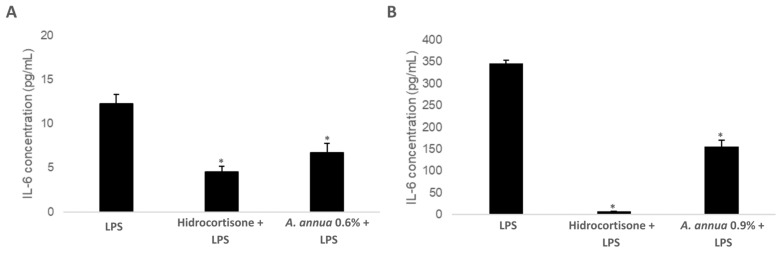
Effect of *Artemisia annua* extract on IL-6 Secretion. RAW 264.7 murine macrophages were stimulated with lipopolysaccharide (LPS) in the presence or absence of either hydrocortisone (positive control) or *Artemisia annua* extracts at two different artemisinin concentrations: 0.6% (**A**) and 0.9% (**B**). IL-6 levels in culture supernatants were quantified and expressed as pg/mL. Bars represent mean ± SD of three independent biological replicates. * *p* < 0.05 compared to the LPS-treated group (Student’s *t*-test).

To evaluate the impact of *Artemisia annua* extracts on IL-6 production, an inflammatory response was induced in RAW 264.7 murine macrophage cells by stimulation with lipopolysaccharide (LPS, 1 µg/mL), a potent endotoxin derived from Gram-negative bacterial walls. Cells were subsequently treated under four conditions: (i) LPS alone, serving as the positive inflammation control; (ii) LPS in combination with *Artemisia annua* extract containing 0.6% artemisinin (0.6% AN); (iii) LPS plus *Artemisia annua* extract containing 0.9% artemisinin (0.9% AN); and (iv) LPS plus hydrocortisone (100 µM), a glucocorticoid with well-established anti-inflammatory properties, used as a positive pharmacological reference control (Figure 3A,B).

Before performing immunomodulatory assays, cytotoxicity tests were conducted on RAW264.7 murine macrophages to determine the optimal working concentration of *Artemisia annua* extracts. Ethanol and acetone extracts containing 0.6% and 0.9% artemisinin, respectively, were evaluated. A 35% (*v*/*v*) concentration was identified as the highest non-cytotoxic dose across both extraction methods and was selected for all subsequent assays.

Under these experimental conditions, IL-6 secretion levels were quantified to determine the anti-inflammatory potential of the *Artemisia annua* extracts. Comparative analysis of the treatment groups provided insights into the dose-dependent effects of artemisinin-enriched extracts on cytokine modulation. The results showed that, under LPS-induced stimulation, treatment with the *Artemisia annua* extract containing 0.6% artemisinin resulted in a moderate decrease in IL-6 secretion, corresponding to a 45.26% inhibition compared to the LPS-only group (Figure 3A). Remarkably, increasing the artemisinin content to 0.9% significantly enhanced the anti-inflammatory response, achieving a 55.16% reduction in IL-6 levels (Figure 3B).

These findings indicate that *Artemisia annua* extracts, particularly with higher artemisinin concentrations, have the potential to modulate the inflammatory response by attenuating IL-6 secretion. This immunoregulatory effect may contribute to a more balanced immune response, enhancing pathogen clearance while reducing the risk of excessive, tissue-damaging inflammation.

### 3.5. Evaluation of the Immunomodulatory Effect of Artemisia annua Extracts: Impact on TNF-α Production

Tumor Necrosis Factor-alpha (TNF-α) is another key pro-inflammatory cytokine involved in both acute and chronic phases of the inflammatory responses. Predominantly secreted by activated macrophages, TNF-α plays a central role in initiating the early phase of inflammation by promoting vascular permeability, inducing apoptosis of infected or damaged cells, and acting synergistically with other inflammatory mediators such as IL-1 and IL-6 [48,49,50].

**Figure 4:**
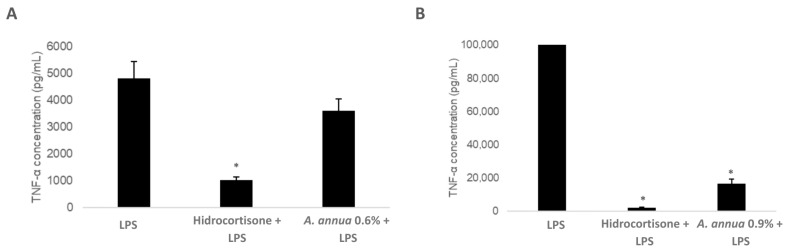
Effect of *Artemisia annua* extract on TNF-α Secretion. RAW 264.7 murine macrophages were stimulated with lipopolysaccharide (LPS) in the presence or absence of either hydrocortisone (positive control) or *Artemisia annua* extracts at two different artemisinin concentrations: 0.6% (**A**) and 0.9% (**B**). TNF-α levels in culture supernatants were quantified and expressed as pg/mL. Bars represent mean ± SD of three independent biological replicates. * *p* < 0.05 compared to the LPS-treated group (Student’s *t*-test).

Following the same experimental design applied in the IL-6 assays, the effect of *Artemisia annua* extracts on TNF-α production was evaluated under inflammatory conditions. RAW 264.7 murine macrophages were stimulated with lipopolysaccharide (LPS, 1 µg/mL) to induce an inflammatory response and then treated with *Artemisia annua* extracts containing either 0.6% or 0.9% artemisinin. Hydrocortisone (100 µM) was used as a positive anti-inflammatory control (Figure 4A,B).

Upon LPS stimulation, treatment with the 0.6% artemisinin extract (0.6% AN) led to a moderate anti-inflammatory response, with a 25.00% reduction in TNF-α levels compared to the LPS-only group (Figure 4A); however, this effect did not reach statistical significance. In contrast, the extract containing 0.9% artemisinin (0.9% AN) produced a markedly greater and statistically significant suppression of TNF-α secretion, achieving an 83.20% reduction (Figure 4B).

These results are consistent with IL-6 results and reinforce the notion that *Artemisia annua* extracts exert artemisinin-dose-dependent anti-inflammatory effects under infection-induced inflammatory stress. This enables the immune system to function more efficiently, effectively targeting pathogens or damaged cells while limiting the risk of collateral inflammatory damage.

## 4. Discussion

*Artemisia annua* is a widely known medicinal plant with a broad therapeutic potential, including anti-tumoral, anti-microbial, anti-parasitic, and immunomodulatory effects [51,52]. Its leishmanicidal activity has been reported against species, such as *Leishmania donovani* and *Leishmania panamensis* [26,27,53]; however, no studies have investigated *its in vitro* effects against *Leishmania infantum*. This parasite strain is the main causative agent of canine leishmaniasis worldwide, particularly in endemic regions such as Southern Europe, Latin America, North Africa, and the Middle East. Our results demonstrate that *Artemisia annua* exhibits in vitro activity against *Leishmania infantum*. This antiparasitic effect was observed in both promastigote (extracellular, infective form transmitted by the sandfly vector) and amastigote (intracellular, macrophage-resident) stages.

Our findings in promastigotes revealed a clear correlation between artemisinin content and anti-leishmanial efficacy. The *Artemisia annua* extract with 1.21% artemisinin exhibited the lowest IC_50_ values, followed by the 0.75% extract, confirming that higher artemisinin levels are associated with increased parasite inhibition. Conversely, extracts with lower artemisinin content showed reduced activity, supporting the hypothesis that artemisinin is the main active component responsible for the leishmanicidal effects. The strong anti-amastigote activity of the 1.21% extract is particularly relevant, as amastigotes are the pathogenic intracellular form in vertebrate hosts. Moreover, and importantly, none of the extracts exhibited cytotoxicity toward J774A.1 murine macrophages at concentrations up to 400 µg/mL, thereby demonstrating selective toxicity against the parasite and supporting a preliminary favorable safety profile in vitro.

Although reference drugs such as meglumine antimoniate display lower IC_50_ values, their well-documented adverse effects limit long-term use and clinical applicability [54,55]. By contrast, *Artemisia annua* extracts, despite requiring higher concentrations (IC_50_ = 69.32 ± 12.79 µg/mL), may represent a safer and more sustainable alternative, especially in chronic cases or patients with comorbidities. Nevertheless, cross-study comparisons should be interpreted cautiously, as IC_50_ values can be influenced by factors such as parasite strains, host cell type, culture conditions, and incubation time [56].

Moreover, previous studies indicate that compounds present in *Artemisia annua* essential oil, including camphor, β-caryophyllene, 1,8-cineole, β-caryophyllene oxide, and β-farnesene, can also reduce parasite load and inflammation in *Leishmania donovani*-infected BALB/c mice [26]. Although artemisinin is recognized as the primary antiparasitic compound, these additional bioactive compounds can also display intrinsic antileishmanial activity and may act synergistically to enhance efficacy compared with artemisinin alone. Furthermore, the pharmacokinetic limitations of isolated artemisinin, such as poor oral absorption and limited bioavailability in canine models, may restrict its therapeutic effectiveness [57]. Whole-plant preparations containing the full phytocomplex offer advantages, as synergistic co-metabolites improve artemisinin absorption and modulate its bioavailability, supporting the use of the complete plant matrix over the isolated compound [58,59].

Given the central role of the immunity in *Leishmania* infection and the reported emerging immunomodulatory properties of *Artemisia annua*, we evaluated its impact on IL-6 and TNF-α levels. These cytokines are among the most prominent pro-inflammatory mediators and are commonly measured in several studies [60,61,62]. During *Leishmania* infection, they are essential for parasite control through macrophage activation and nitric oxide production, but sustained overexpression contributes to tissue damage and disease severity [49]. In light of their role in pathological outcomes, these cytokines were therefore selected for measurement in the present study. Notably, *Artemisia annua* has been reported to modulate immune polarization by downregulating Th2 responses and promoting a Th1-type profile, in which a balanced interplay of pro- and anti-inflammatory mediators is crucial for eliminating intracellular pathogens [26,36].

By suppressing excessive pro-inflammatory cytokines, *Artemisia annua* helps resolve inflammation, promotes a return to immune homeostasis, and mitigates collateral tissue damage. Indeed, persistent elevation of pro-inflammatory cytokines has been linked not only to leishmaniasis, but also to numerous chronic inflammatory and autoimmune diseases, such as cardiovascular disorders, type 2 diabetes, rheumatoid arthritis and neurodegenerative conditions [63,64,65]. Thus, downregulating these mediators represents a strategic approach to fine-tune immune responses. Phytochemicals with such activity, including *Artemisia annua*, may improve host tolerance to infection and clinical outcomes by limiting inflammatory toxicities and cytokine release syndrome (CRS) observed in severe infections, cancer, and some immunotherapies.

During *Leishmania* infection, particularly with *Leishmania infantum*, macrophages act as the main host cells for parasite replication. Normally, apoptosis of infected macrophages limits intracellular survival, but in leishmaniasis this process is impaired. A key contributing factor to this impairment is the pronounced elevation of pro-inflammatory cytokines (TNF-α, IL-6, IL-1β). Although these cytokines are typically associated with antimicrobial defense, their excessive and sustained production can paradoxically contribute to macrophage survival rather than elimination [66,67].

Elevated pro-inflammatory signaling can activate NF-κB and other survival pathways within macrophages, increasing anti-apoptotic proteins (Bcl-2, Bcl-xL) [68,69]. This may facilitate the recruitment of additional monocytes, which can differentiate into new macrophages and serve as new cellular targets for infection, thereby perpetuating the parasitic cycle. Such immune evasion strategy facilitates chronic intracellular persistence and complicates parasite clearance without therapeutic intervention [70,71,72].

Interestingly, our assays showed that *Artemisia annua* effectively attenuates pro-inflammatory cytokine levels under inflammatory conditions, thereby enhancing the immune system to function more efficiently, enhancing its ability to target pathogens or damaged cells while minimizing the risk of inflammatory collateral tissue damage.

It is important to emphasize that these immunological assays were performed in LPS-stimulated RAW macrophages in the absence of *Leishmania* infection. Therefore, the observed reduction in IL-6 and TNF-α levels represents a direct immunomodulatory effect of *Artemisia annua* on macrophages. These findings highlight the intrinsic capacity of the plant to modulate inflammatory responses; however, caution is warranted when extrapolating to infection contexts, as cytokine regulation in *Leishmania*-infected macrophages may differ. Future studies will be required to confirm whether similar effects occur under parasitic stimulation and to clarify the relative contributions of parasite inhibition and host immunomodulation. In addition, further investigations should evaluate other cytokines and immunomodulatory markers to broaden the understanding of the underlying mechanisms.

Consistent with its antiparasitic performance, a clear dose-dependent relationship was observed between artemisinin concentration and immunomodulatory effects. Nevertheless, these results should be interpreted carefully, as RAW macrophages were used for the immune response experiments and J774 macrophages for the toxicity assays against amastigote forms. RAW 264.7 cells are commonly used for testing immunomodulatory activity after stimulation with LPS, a component of Gram-negative bacteria. However, as the immune response induced by *Leishmania infantum* may differ, our findings only demonstrate the activity of *Artemisia annua* in LPS-induced macrophages. This represents a limitation that should be acknowledged given the variability of immune responses across macrophage cell lines and between parasitic infection and bacterial stimulation. At the same time, the use of two distinct macrophage lines under different conditions broadens the scope of the study by providing complementary insights into the effects of *Artemisia annua* across different cellular contexts.

**Figure 5:**
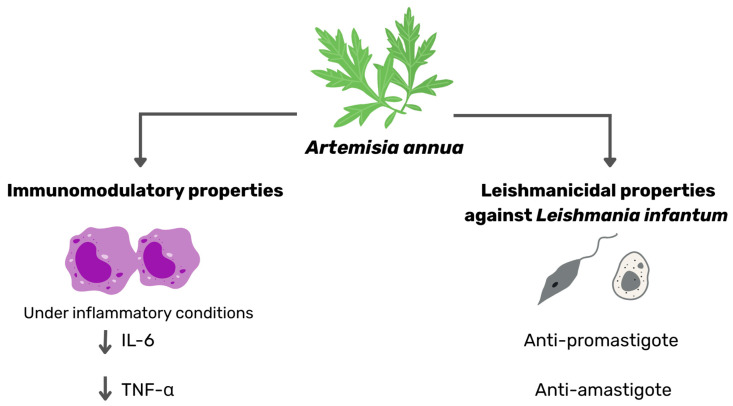
Schematic overview of the main findings illustrating the antiparasitic effects of *Artemisia annua* against *Leishmania infantum* and its associated immunomodulatory activity.

Medicinal plants contain diverse bioactive compounds, but without rigorous standardization, their variable composition may lead to inconsistent clinical outcomes, reduced efficacy, or even safety issues. Standardization is thus essential to guarantee quality, safety, reproducibility, and clinical acceptance [73,74,75,76,77,78,79]. In *Artemisia annua*, artemisinin is the primary bioactive molecule [80], yet its concentration can vary greatly depending on chemotype, cultivation parameters (soil composition, climate, photoperiod), harvest timing (optimal during floral induction), and post-harvest processes such as drying and storage conditions [81,82,83,84,85]. Our findings highlight the dual role of artemisinin in exhibiting both antileishmanial and immunomodulatory properties (see summary in Figure 5), underscoring the need to standardize its plant content to ensure therapeutic potency and reliable efficacy.

Indeed, evaluations of veterinary *A. annua* supplements in the U.S. revealed major discrepancies, with none meeting label claims for artemisinin content and one containing no detectable artemisinin [86]. These findings reinforce the urgent need for robust standardization practices, positioning artemisinin as the key quality marker to safeguard product integrity, protect patient health, and secure therapeutic reliability.

## 5. Conclusions

This study provides the first in vitro evidence that *Artemisia annua* extracts exert leishmanicidal activity against *Leishmania infantum*, the main etiological agent of canine leishmaniasis. Activity was demonstrated in both promastigote and amastigote stages, with extracts richer in artemisinin showing the greatest efficacy, reinforcing its role as the principal bioactive component. These results position artemisinin-rich *Artemisia annua* extracts as promising candidates for the development of novel plant-based therapies, especially given the limitations of current therapies.

In parallel to antiparasitic activity, *Artemisia annua* displayed immunomodulatory effects in an artemisinin-dose-dependent manner, attenuating excessive pro-inflammatory cytokines and promoting a balanced Th1 response. This dual mechanism, direct parasite inhibition and immune regulation, highlights its potential not only for leishmaniasis but also for other intracellular infections and inflammatory disorders.

The therapeutic potential of *Artemisia annua* depends on rigorous standardization particularly of artemisinin content, to ensure safety, efficacy, and reproducibility. Variability in phytochemical composition remains a major limitation; thus, standardized production is essential for future clinical development.

In summary, standardized, artemisinin-rich *A. annua* extracts emerge as promising effective adjuvant phytotherapeutic candidates against canine leishmaniasis warranting further in vivo studies and clinical validation.

## Data Availability

Data are contained within the article and Supplementary Material. Further inquiries can be directed to the corresponding author.

## Supplementary Materials

The following supporting information can be downloaded at: https://www.mdpi.com/article/10.3390/vetsci12100950/s1, Figure S1: Activity of Artemisia annua extract containing 0.13% artemisinin.
