# Supplementary material for: Standardized Artemisia annua Exhibits Dual Antileishmanial Activity and Immunomodulatory Potential In Vitro
Source: Vet Sci. 2025 Oct 1;12(10):950. doi: 10.3390/vetsci12100950 (PMC12568091; doi:10.3390/vetsci12100950)
Supplement: Supplementary file 1 [file vetsci-12-00950-s001.zip › Supplementary Figure S1.pdf]

A

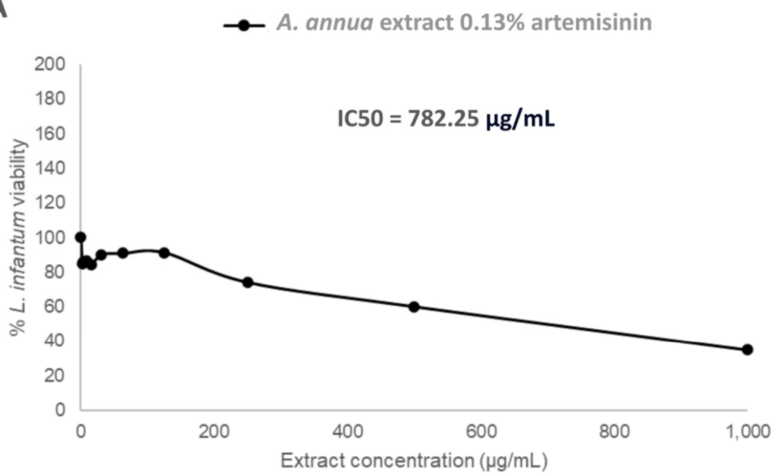

B

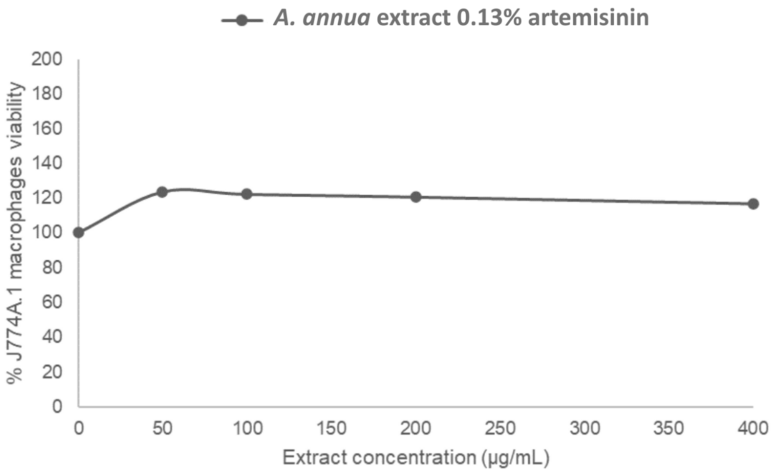

Supplementary Figure S1. Activity of *Artemisia annua* extract containing 0.13% artemisinin.

(A) Anti-promastigote activity of *Artemisia annua* leaves. Log-phase *Leishmania infantum* promastigotes were incubated for 72 h at 27 °C with an *Artemisia annua* extracts containing 0.13% artemisinin. Promastigote viability was assessed by a colorimetric assay using Alamar Blue®, following exposure to serial dilutions of each extract (1 mg/mL to 1.95 µg/mL). IC_{50} values was determined. One independent experiment was performed.

(B) Cytotoxicity assay on uninfected J774A.1 macrophages treated with increasing concentrations of the same extract (50-400 µg/mL) for 24 h. Cell viability was measured using AlamarBlue® and remained above 90% at all tested concentrations.
